# Outcomes of patients with acute liver failure not listed for liver transplantation: A cohort analysis

**DOI:** 10.1097/HC9.0000000000000575

**Published:** 2024-10-30

**Authors:** Victor Dong, Valerie Durkalski, William M. Lee, Constantine J. Karvellas, and the United States Acute Liver Failure Study Group

**Affiliations:** 1 Department of Critical Care Medicine, University of Calgary, Calgary, Alberta, Canada; 2 Department of medicine, Division of Gastroenterology, University of Calgary, Calgary, Alberta, Canada; 3 Department of Public Health Sciences, Medical University of South Carolina, Charleston, South Carolina, USA; 4 Department of medicine, Division of Digestive and Liver Diseases, University of Texas Southwestern Medical Center at Dallas, Dallas, Texas, USA; 5 Department of Critical Care Medicine, University of Alberta, Edmonton, Alberta, Canada; 6 Department of medicine, Division of Gastroenterology (Liver Unit), University of Alberta, Edmonton, Alberta, Canada

**Keywords:** acute liver failure, ALFSG prognostic index, liver transplantation, prognosis

## Abstract

**Background:**

Acute liver failure (ALF) is a rare condition leading to morbidity and mortality. Liver transplantation (LT) is often required, but patients are not always listed for LT. There is a lack of data regarding outcomes in these patients. Our aim is to describe outcomes of patients with ALF not listed for LT and to compare this with those listed for LT.

**Methods:**

Retrospective analysis of all nonlisted patients with ALF enrolled in the Acute Liver Failure Study Group (ALFSG) registry between 1998 and 2018. The primary outcome was 21-day mortality. Multivariable logistic regression was done to identify factors associated with 21-day mortality. The comparison was then made with patients with ALF listed for LT.

**Results:**

A total of 1672 patients with ALF were not listed for LT. The median age was 41 (IQR: 30–54). Three hundred seventy-one (28.9%) patients were too sick to list. The most common etiology was acetaminophen toxicity (54.8%). Five hundred fifty-eight (35.7%) patients died at 21 days. After adjusting for relevant covariates, King’s College Criteria (adjusted odds ratio: 3.17, CI 2.23–4.51), mechanical ventilation (adjusted odds ratio: 1.53, CI: 1.01–2.33), and vasopressors (adjusted odds ratio: 2.10, CI: 1.43–3.08) (*p* < 0.05 for all) were independently associated with 21-day mortality. Compared to listed patients, nonlisted patients had higher mortality (35.7% vs. 24.3%). Patients deemed not sick enough had greater than 95% survival, while those deemed too sick still had >30% survival.

**Conclusions:**

Despite no LT, the majority of patients were alive at 21 days. Survival was lower in nonlisted patients. Clinicians are more accurate in deeming patients not sick enough to require LT as opposed to deeming patients too sick to survive.

## INTRODUCTION

Acute liver failure (ALF) is a rare syndrome that occurs following a severe insult to hepatocytes in a patient without prior underlying chronic liver disease.^1^ Within at least 26 weeks of the insult, often much less, rapid deterioration of hepatic function results in jaundice, coagulopathy, and HE followed by multiorgan failure (MOF).^2^ ALF is associated with significant morbidity and mortality. Clinical outcomes generally depend on the etiology of ALF and range from spontaneous recovery to the need for emergent liver transplantation (LT) to mortality.^3^ Overall, mortality is around 30%.^4^,^5^

Treatment of ALF is mainly supportive and aims to prevent and control cerebral edema, correct metabolic derangements, and maintain hemodynamic stability.^6^,^7^ Over the past 2 decades, ALF outcomes have steadily improved, especially for acetaminophen (APAP)-induced ALF.^8^ This is mainly due to improved recognition and intensive care unit therapies. Prompt *N*-acetylcysteine usage for both APAP and non-APAP–induced ALF, early initiation of continuous renal replacement therapy (CRRT), and use of high-volume plasma exchange have all improved survival in patients with ALF.^9^,^10^,^11^,^12^,^13^

However, a number of patients fail to recover with maximal medical therapies and require emergent LT. Despite being critically ill at the time of transplant, 1-year and 3-year post-LT survival is still as high as 91% and 90%, respectively.^4^ Even when early consideration of LT occurs, certain factors may preclude patients from being listed for LT, including severe MOF and a variety of psychosocial factors.^14^,^15^

Currently, there is a paucity of epidemiologic data on outcomes of patients with ALF not listed for LT. Therefore, our primary objective was to analyze prospectively collected ALF patient data from the multicenter Acute Liver Failure Study Group (ALFSG) registry between 1998 and 2018 to evaluate factors leading to nonlisting of patients with ALF, outcomes of these patients, including 21-day survival, and lastly, factors associated with mortality in nonlisted patients with ALF. We then compared outcomes of nonlisted patients with ALF to those who were listed for LT to determine how accurately patients deemed either too well or too sick for LT are prognosticated.

## METHODS

### Study design

We performed a retrospective cohort study of all patients with ALF prospectively enrolled in the ALFSG registry between January 1998 and December 2018 who were evaluated but not listed for LT (n = 1672). All patients not listed for LT had an evaluation for LT, and none passed away before LT waitlist decision. We included all nonlisted patients with outcomes data (n = 1564). We then added patients listed for LT (n = 934) to our study for comparison to nonlisted patients. A detailed report of the listed patients in this same registry has been previously published.^16^ All respective institutional review boards/health research ethics boards at participating sites (tertiary LT referral centers) within the ALFSG provided approval for the study’s protocol. Written informed consent was obtained from each participant or next of kin (in cases of HE at the time of enrollment). All research procedures were conducted according to the principles of the 1975 Declaration of Helsinki. Therapeutic interventions and monitoring were implemented according to participating institutional standards of care. Criteria for listing and performing LT were those utilized at participating centers. This study was written according to the STROBE guideline for reporting retrospective studies.^17^

### Participants

Inclusion criteria were as follows: (1) evidence of ALF according to the enrollment criteria of the ALFSG (see operational definitions), (2) participant age ≥18 years, and (3) not listed for LT. Patients listed for liver transplants were also included to allow comparison to nonlisted patients. Exclusion criteria were as follows: (1) evidence of cirrhosis/acute-on-chronic liver failure and (2) severe acute liver injury only defined by the presence of coagulopathy with an international normalized ratio (INR) ≥1.5, illness onset <26 weeks from hepatic injury, and no presence of HE.

### Operational definitions

ALF was defined by the following criteria: (1) HE of any degree (West Haven Criteria), (2) evidence of coagulopathy with an INR ≥1.5, (3) illness onset <26 weeks from hepatic injury, and (4) no evidence of cirrhosis. The King’s College Criteria (KCC) qualifies poor prognosis in ALF: for APAP-induced ALF, KCC is defined as either (1) arterial pH <7.3, or (2) all 3 of (i) INR >6.5, (ii) creatinine >300 μmol/L (3.4 mg/dL), and (iii) the presence of grade 3/4 HE; for non-APAP–induced ALF, KCC is defined as either (1) INR >6.5, or (2) 3 of (i) poor prognosis etiology, (ii) time from jaundice to HE >7 days, (iii) age<10 or >40, iv) INR >3.5, and (v) serum bilirubin >17 mg/dL.^18^ The Acute Liver Failure Study Group Prognostic Index (ALFSG-PI) is an internally validated mathematical model that predicts 21-day transplant-free survival (TFS) of patients with ALF using hospital admission data and it has been previously described.^19^ The MELD is calculated as follows: [3.78 × ln(bilirubin in mg/dL) + 11.2 × ln(INR) + 9.57 × ln(creatinine in mg/dL) + 6.43]; a serum creatinine value of 354 μmol/L (4 mg/dL) is substituted for dialyzed patients.^20^ Renal replacement therapy (RRT) included both intermittent hemodialysis and CRRT. Patients receiving CRRT and intermittent hemodialysis during days 1–7 were coded accordingly.

### Data collection

Patients were enrolled prospectively into the ALFSG registry (data coordinating centers at the University of Texas Southwestern Medical Center, 1998–2010 and the Medical University of South Carolina, 2010–2018) and analyzed retrospectively. Detailed demographic, clinical, and outcome data on patients with ALF were recorded in an electronic database with the observation period beginning at the time of study enrollment. Baseline data on those with ALF who were not listed for LT and those who were listed for LT included demographic features, etiology of ALF, blood biochemistries, HE grade, and MELD score. Additional data including organ support requirements (invasive mechanical ventilation and oxygenation status [PaO_2_/FiO_2_]), vasopressors, and RRT were recorded. Clinical outcomes included intensive care unit complications, causes of death, and overall 21-day survival. The primary outcome was overall 21-day survival.

### Statistical analysis

Statistical analysis was performed using Stata (version 18.0; StataCorp). Continuous variables were reported as medians with IQR following testing for normality and compared using the Student *t* test (normal distribution) and Wilcoxon Rank sum test (nonparametric variables). Categorical variables were reported as numbers and percentages and were compared using the chi-squared test. To study the association between overall 21-day survival and nonlisting for ALF, multivariable logistic regression analyses were performed. Covariates were included based on their significance in the univariable regression analysis (*p* < 0.20). Collinear variables were excluded where appropriate. Results were reported as adjusted odds ratios (aORs) and 95% CIs. Model performance was assessed using the c-statistic (AUROC). We used a statistical significance threshold of 0.05 for 2-tailed *p* values.

## RESULTS

### Baseline characteristics of nonlisted patients

In total, 1672 patients (64%) with ALF who were not listed for LT at any time were identified in the ALFSG registry. Out of those, 1564 had data on survival. Median (IQR) age was 42 (30–54) years, and 497 (31.8%) were male. Around half of the patients (51.4%) were enrolled in the recent era (2009–2018). APAP was the etiology of ALF in 843 (53.9%) patients. Reasons recorded for not listing for LT included patients not being sick enough (33.3%), patients having psychosocial contraindications (28.4%), as well as patients being too sick (29.8%), which included irreversible brain injury (1.6%) and sepsis (3.0%). Nonlisted patients had median MELD of 36 (26–44) and ALFSG-PI of 36.8% (12.4%–69.0%), with 948 (61.8%) patients having high-grade HE and 324 (20.7%) patients meeting KCC. Regarding organ supports, 905 (57.9%) nonlisted patients required mechanical ventilation, 540 (34.5%) patients needed vasopressor support, and 520 (33.3%) patients required RRT, with 244 (15.6%) receiving CRRT. Demographic data for nonlisted patients with ALF are described in Table 1.

**TABLE 1:** Demographics and outcomes of 1564 ALF patients not listed for LT

	Overall (N = 1564)	
	N	Number (%) or median (IQR)
Age	1564	42 (30–54)
Year of enrollment		
1998–2008	1564	760 (48.6)
2009–2018	1564	804 (51.4)
Sex (male)	1564	497 (31.8)
Reason declined for LT		
Not sick enough	1217	405 (33.3)
Psychosocial	1217	346 (28.4)
Too sick	1217	362 (29.8)
Irreversible brain injury		20 (1.6)
Sepsis		37 (3.0)
Etiology		
APAP	1564	843 (53.9)
DILI	1564	130 (8.3)
HBV	1564	80 (5.1)
AIH	1564	54 (3.5)
Indeterminate	1564	122 (7.8)
Other	1564	90 (5.8)
King’s College Criteria	1564	324 (20.7)
Highest MELD (during 7 d)	1550	36 (26–44)
Coma Grade 3/4 (highest during days 1–7)	1535	948 (61.8)
ALFSG Prognostic Index (highest days 1–7)	1521	36.8% (12.4%–69.0%)
Organ support (days 1–7)		
Mechanical ventilation	1564	905 (57.9)
Vasopressors	1564	540 (34.5)
Renal replacement therapy	1564	520 (33.3)
Hemodialysis	1564	334 (21.4)
CVVH	1564	244 (15.6)
Admission biochemistry		
Hemoglobin (g/dL)	1542	10.8 (9.4–12.3)
White blood cells (10^9^/L)	1541	9.8 (6.6–14.6)
Platelets (10^9^/L)	1534	119 (74–179)
INR	1526	2.6 (1.9–4.0)
ALT (IU/L)	1530	2344 (830–4620)
Bilirubin (mg/dL)	1532	5.3 (3.0–11.2)
pH	1152	7.41 (7.34–7.47)
Ammonia (venous) (μmol/L)	592	89 (58–140)
Creatinine (mg/dL)	1548	1.8 (0.9–3.2)
Lactate (mmol/L)	911	3.7 (2.2–7.2)
Phosphate (mg/dL)	1336	3 (2.0–4.4)
ICP therapies (days 1–7)		
ICP monitor	1474	79 (5.4)
Mannitol	1564	197 (12.6)
Barbiturate	1564	76 (4.9)
Hypothermia	1564	71 (4.5)
Sedatives	1564	902 (57.7)
Blood products (days 1–7)		
Red blood cells	1564	433 (27.7)
Fresh frozen plasma	1564	640 (40.9)
Platelets	1564	272 (17.4)
ICU complications (days 1–7)		
Seizures	1564	86 (5.5)
Arrhythmia	1564	340 (21.7)
Gastrointestinal bleeding	1564	144 (9.2)
Abnormal chest x-ray	844	556 (65.9)
Bacteremia/blood stream infection	1564	227 (14.5)
Intra-study NAC		
i.v.	1564	1029 (65.8)
Oral	1564	658 (42.1)
Psychiatric comorbidities	1564	645 (41.2)
Overdose intent (IF APAP)		
Suicide attempt	897	327 (36.5)
Unintentional	897	454 (50.6)
Unknown	897	116 (12.9)
Alcohol use		
<7 drinks/wk	638	463 (72.6)
>=7 drinks/wk	638	175 (27.4)
Intravenous drug use	1549	119 (7.7)
Death (days 1–21)	1564	558 (35.7)
Cause of death		
Multiorgan failure	1297	235 (18.1)
Cerebral edema	1297	80 (6.2)
Unknown	1297	84 (6.5)
Outcome (day 21)		
Survival	1564	1006 (64.3)
Death	1564	558 (35.7)

Abbreviations: AIH, autoimmune hepatitis; ALF, acute liver failure; ALFSG, Acute Liver Failure Study Group; APAP, acetaminophen; CVVH, continuous veno-venous hemofiltration; ICP, intracranial pressure; ICU, intensive care unit; INR, international normalized ratio; i.v., intravenous; LT, liver transplantation; NAC, *N*-acetylcysteine.

### Outcomes of nonlisted patients

Death at 21 days post-enrollment occurred in 558 (35.7%) of nonlisted patients, with 235 (18.1%) patients dying from MOF and 80 (6.2%) patients dying from cerebral edema. When comparing nonlisted survivors to nonsurvivors, survivors were younger (39 vs. 46 y of age, *p* < 0.001), had higher APAP etiology rates (63.1% vs. 37.3%, *p* < 0.001), lower KCC rates (9.2% vs. 41.4%, *p* < 0.001), lower high-grade HE rates (48.8% vs. 85.2%, *p* < 0.001), and had higher ALFSG-PI (53.9% vs. 11.4%, *p* < 0.001). Nonlisted ALF survivors required less mechanical ventilation (46.2% vs. 78.9%, *p* < 0.001), less vasopressor use (19.5% vs. 61.6%, *p* < 0.001), and less RRT (28.7% vs. 41.4%, *p* < 0.001). Survivors were more likely to not be listed for not being sick enough (48.6% vs. 4.1%, *p* < 0.001) and less likely to not be listed for being too sick (15.5% vs. 56.8%, *p* < 0.001). Of the patients who died, 4.1% were deemed not sick enough to list for LT. Of the patients who survived, 15.5% were considered too sick to survive. A comparison of nonlisted ALF survivors to nonsurvivors is described in Table 2.

**TABLE 2:** Demographics of 1564 patients with ALF not listed for LT stratified based on overall 21-day survival

	Alive at day 21 (N = 1006)		Deceased at day 21 (N = 558)		
	N		N		*p*
Age	1006	39 (29–51)	558	46 (34–59)	<0.001
Year of enrollment	1006		558		0.104
1998–2008	1006	475 (47.2%)	558	285 (51.1%)	0.144
2009–2018	1006	531 (52.8%)	558	273 (48.9%)	0.144
Sex (male)	1006	294 (29.2%)	558	203 (36.4%)	0.004
Reason declined for LT					
Not sick enough	798	388 (48.6%)	419	17 (4.1%)	<0.001
Psychosocial	798	240 (30.1%)	419	106 (25.3%)	0.079
Too sick	798	124 (15.5%)	419	238 (56.8%)	<0.001
Irreversible brain injury	798	1 (0.1%)	419	19 (4.5%)	<0.001
Sepsis	798	13 (1.6%)	419	24 (5.7%)	<0.001
Etiology (we will get all the etiology information)					
APAP	1006	635 (63.1%)	558	208 (37.3%)	<0.001
DILI	1006	71 (7.1%)	558	59 (10.6%)	0.016
HBV	1006	29 (2.9%)	558	51 (9.1%)	<0.001
AIH	1006	23 (2.3%)	558	31 (5.6%)	<0.001
Indeterminate	1006	54 (5.4%)	558	68 (12.2%)	<0.001
Other	1006	28 (2.8%)	558	62 (11.1%)	<0.001
King’s College Criteria	1006	93 (9.2%)	558	231 (41.4%)	<0.001
Highest MELD (during 7 d)	1003	32 (23–40)	547	42 (34–49)	<0.001
Coma Grade 3/4 (highest during days 1–7)	988	482 (48.8%)	547	466 (85.2%)	<0.001
ALFSG Prognostic Index (highest days 1–7)	985	53.9% (29.7%–80.7%)	536	11.4% (4.3%–26.0%)	<0.001
Organ support (days 1–7)					
Mechanical ventilation	1,006	465 (46.2%)	558	440 (78.9%)	<0.001
Vasopressors	1,006	196 (19.5%)	558	344 (61.6%)	<0.001
Renal replacement therapy	1,006	289 (28.7%)	558	231 (41.4%)	<0.001
Hemodialysis	1,006	197 (19.6%)	558	137 (24.6%)	0.022
CVVH	1,006	131 (13.0%)	558	113 (20.3%)	<0.001
Admission biochemistry					
Hemoglobin (g/dL)	991	10.9 (9.5–12.6)	551	10.4 (9.1–12.0)	<0.001
White blood cells (10^9^/L)	991	9.3 (6.4–13.6)	550	11.0 (7.3–16.6)	<0.001
Platelets (10^9^/L)	986	127 (85–185)	548	103 (60–160)	<0.001
INR	984	2.4 (1.8–3.5)	542	3.1 (2.3–4.8)	<0.001
ALT (IU/L)	988	2701 (1278–4997)	542	1423 (468–3971)	<0.001
Bilirubin (mg/dL)	986	4.5 (2.4–7.9)	546	8.6 (4.1–20.1)	<0.001
pH	728	7.42 (7.36–7.47)	424	7.39 (7.29–7.46)	<0.001
Ammonia (venous) (μmol/L)	388	80 (55–122)	204	112 (72–174)	<0.001
Creatinine (mg/dL)	997	1.5 (0.8–3.0)	551	2.3 (1.3–3.6)	<0.001
Lactate (mmol/L)	583	2.7 (1.8–4.8)	328	6.8 (4.0–11.7)	<0.001
Phosphate (mg/dL)	864	2.7 (1.8–3.7)	472	4.0 (2.7–5.9)	<0.001
ICP therapies (days 1–7)					
ICP monitor	953	41 (4.3%)	521	38 (7.3%)	0.015
Mannitol	1006	78 (7.8%)	558	119 (21.3%)	<0.001
Barbiturate	1006	29 (2.9%)	558	47 (8.4%)	<0.001
Hypothermia	1006	34 (3.4%)	558	37 (6.6%)	0.003
Sedatives	1006	516 (51.3%)	558	386 (69.2%)	<0.001
Blood products (days 1–7)					
Red blood cells	1006	227 (22.6%)	558	206 (36.9%)	<0.001
Fresh frozen plasma	1006	317 (31.5%)	558	323 (57.9%)	<0.001
Platelets	1006	129 (12.8%)	558	143 (25.6%)	<0.001
ICU complications (days 1–7)					
Seizures	1006	32 (3.2%)	558	54 (9.7%)	<0.001
Arrhythmia	1006	177 (17.6%)	558	163 (29.2%)	<0.001
Gastrointestinal bleeding	1006	61 (6.1%)	558	83 (14.9%)	<0.001
Abnormal chest x-ray	473	282 (59.6%)	371	274 (73.6%)	<0.001
Bacteremia/blood stream infection	1006	148 (14.7%)	558	79 (14.2%)	0.766
Intra-study NAC					
i.v.	1006	699 (69.5%)	558	330 (59.1%)	<0.001
Oral	1006	458 (45.5%)	558	200 (35.8%)	<0.001
Psychiatric comorbidities	1006	467 (46.4%)	558	178 (31.9%	<0.001
Overdose intent (IF APAP)					
Suicide attempt	586	245 (41.8%)	195	82 (42.1%)	0.953
Unintentional	586	341 (58.2%)	195	113 (58.0%	0.953
Unknown	659	73 (11.1%)	238	43 (18.1%)	0.006
Alcohol use					
<7 drinks/wk	428	302 (70.6%)	210	161 (76.7%)	0.104
>=7 drinks/wk	428	126 (29.4%)	210	49 (23.3%)	0.104
Intravenous drug use	999	96 (9.6%)	550	23 (4.2%)	<0.001

Abbreviations: AIH, autoimmune hepatitis; ALF, acute liver failure; ALFSG, Acute Liver Failure Study Group; APAP, acetaminophen; CVVH, continuous veno-venous hemofiltration; ICP, intracranial pressure; ICU, intensive care unit; INR, international normalized ratio; i.v., intravenous; LT, liver transplantation; NAC, *N*-acetylcysteine.

### Multivariable analysis of nonlisted patients: Association with mortality at 21 days

**FIGURE 1:**
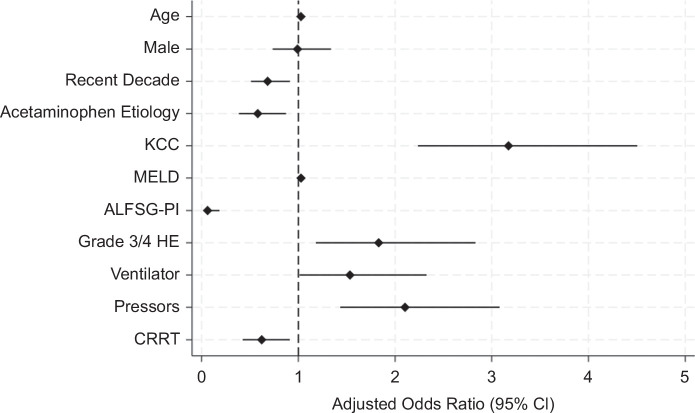
Adjusted independent associations with increased and decreased death at 21 days in nonlisted patients with ALF. Abbreviations: ALFSG-PI, Acute Liver Failure Study Group prognostic index; CRRT, continuous renal replacement therapy; KCC, King’s College criteria.

To determine variables independently associated with mortality at 21 days, multivariable logistic regression analysis was performed. Testing for collinearity did not reveal any collinear variables. In our final multivariate model, after adjusting for covariates, age (aOR: 1.03 [95% CI: 1.02–1.04], *p* < 0.001), KCC (aOR: 3.17 [95% CI: 2.23–4.51], *p* < 0.001), MELD (aOR: 1.03 [95% CI: 1.01–1.05], *p* = 0.004), coma (high-grade HE) (aOR: 1.83 [95% CI: 1.18–2.83], *p* = 0.007), mechanical ventilation (aOR: 1.53 [95% CI: 1.01–2.33], *p* = 0.043), and vasopressor use (aOR: 2.10 [95% CI: 1.43–3.08], *p* < 0.001) were all independently associated with mortality at 21 days. Recent era (aOR: 0.68 [95% CI: 0.51–0.91], *p* = 0.01), APAP etiology (aOR: 0.58 [95% CI: 0.38–0.87], *p* = 0.009), ALFSG-PI (aOR: 0.06 [95% CI: 0.02–0.18], *p* < 0.001), and CRRT use (aOR: 0.62 [95% CI: 0.42–0.91], *p* = 0.015) were all independently associated with reduced mortality at 21 days. This is shown in Table 3 and Figure 1. This model performed well with an AUROC of 0.875.

**TABLE 3:** Logistic regression for nonlisted ACLF patients (n=1564)

	Univariate		
Variable	N	Odds ratio	*p*
Age	1564	1.03 (1.02–1.03)	<0.001
Male (vs. female)	1564	1.38 (1.11–1.72)	0.004
Recent era (vs. early era)	1564	0.86 (0.70–1.05)	0.144
APAP etiology (vs Non-APAP etiology)	1564	0.35 (0.28–0.43)	<0.001
King’s College Criteria (days 1–7)	1564	6.94 (5.28–9.10)	<0.001
Highest MELD (days 1–7)	1550	1.08 (1.07–1.10)	<0.001
Highest ALFSG Prognostic Index (days 1–7)	1521	0.004 (0.002–0.008)	<0.001
Coma Grade 3/4 (days 1–7)	1535	6.04 (4.62–7.89)	<0.001
Ventilation (days 1–7)	1564	4.34 (3.41–5.50)	<0.001
Vasopressors (days 1–7)	1564	6.64 (5.27–8.37)	<0.001
CRRT(days 1–7)	1564	1.70 (1.29–2.24)	<0.001
	Multivariable model: N = 1521; AUROC = 0.875		
**Variable**	**Included in model**	**Adjusted odds ratio**	***p***
Age	Yes	1.03 (1.02–1.04)	<0.001
Male (vs. female)	Yes	0.99 (0.73–1.34)	0.953
Recent era (vs. early era)	Yes	0.68 (0.51–0.91)	0.01
APAP etiology (vs Non-APAP etiology)	Yes	0.58 (0.38–0.87)	0.009
King’s College Criteria (days 1–7)	Yes	3.17 (2.23–4.51)	<0.001
Highest MELD (days 1–7)	Yes	1.03 (1.01–1.05)	0.004
Highest ALFSG Prognostic Index (days 1–7)	Yes	0.06 (0.02–0.18)	<0.001
Coma Grade 3/4 (days 1-7)	Yes	1.83 (1.18–2.83)	0.007
Ventilation (days 1–7)	Yes	1.53 (1.01–2.33)	0.043
Vasopressors (days 1–7)	Yes	2.10 (1.43–3.08)	<0.001
CRRT(days 1–7)	Yes	0.62 (0.42–0.91)	0.015

*Note*: Independent associations for death at 21 days. Collinearity testing was completed, showing no collinearity. AUROC is 0.875. Final model: [log(odds of death at 21 days) = −2.512 + 0.026 (Age) – 0.009 (Male) – 0.384 (recent era) – 0.547 (APAP etiology) + 1.156 (KCC) + 0.027 (MELD) – 2.789 (ALFSG-PI) + 0.604 (Coma) + 0.428 (Ventilator) + 0.743 (Vasopressor) – 0.477 (CRRT)].

Abbreviations: ALFSG, Acute Liver Failure Study Group; ALFSG-PI, Acute Liver Failure Study Group Prognostic Index; APAP, acetaminophen; CRRT, continuous renal replacement therapy; KCC, King’s College Criteria.

### Comparison of nonlisted and listed patients

After comparing nonlisted patients with ALF to those listed for LT, nonlisted patients were older (42 vs. 38 y of age, *p* < 0.001), a higher proportion enrolled in the recent era (51.4% vs. 39.0%, *p* < 0.001), were more likely to have APAP etiology (53.9% vs. 29.1%, *p* < 0.001), were less likely to meet KCC (20.7% vs. 28.3%, *p* < 0.001), and had higher ALFSG-PI (36.8% vs. 15.0%, *p* < 0.001). Survival at 21 days was significantly lower (64.3% vs. 75.7%, *p* < 0.001) in nonlisted patients. A comparison between nonlisted and listed patients with ALF is shown in Supplemental Table S1, http://links.lww.com/HC9/B72.

### Comparison of nonlisted (not sick enough) and listed patients

After separating nonlisted patients with ALF into 2 categories (not sick enough and too sick) and then comparing these patients to listed patients, the nonlisted (not sick enough) patients had a higher proportion being enrolled in the recent era (61.2% vs. 39.0%, *p* < 0.001), a higher proportion having APAP etiology (63.7% vs. 29.1%, *p* < 0.001), were less likely to fulfill KCC (5.4% vs. 28.3%, *p* < 0.001), had lower MELD (29 vs. 36, *p* < 0.001), were less likely to have high-grade HE (34.2% vs. 65.4%, *p* < 0.001), and had significantly higher ALFSG-PI (67.6% vs. 15.0%, *p* < 0.001). Survival at 21 days was significantly higher for nonlisted (not sick enough) patients (95.8% vs. 75.7%, *p* < 0.001). Comparison between nonlisted (not sick enough) and listed patients is demonstrated in Supplemental Table S2, http://links.lww.com/HC9/B72.

### Comparison of nonlisted (too sick) and listed patients

When comparing nonlisted (too sick) patients to those who were listed, nonlisted (too sick) patients were older (51 vs. 38 y of age, *p* < 0.001), had higher proportion enrolled in the recent era (70.4% vs. 39.0%, *p* < 0.001), higher MELD (40 vs. 36, *p* < 0.001), higher rates of high-grade HE (75.3% vs. 65.4%, *p* < 0.001), had similar rates of APAP etiology, similar rates of the fulfillment of KCC, and similar ALFSG-PI. Survival at 21 days was significantly lower for nonlisted (too sick) patients (34.4% vs. 75.7%, *p* < 0.001). Comparison between nonlisted (too sick) and listed patients is demonstrated in Supplemental Table S3, http://links.lww.com/HC9/B72.

#### Comparison of nonlisted (not sick enough) survivors and nonsurvivors

Nonlisted patients with ALF deemed not sick enough for LT listing had a 95.8% survival rate. When survivors and nonsurvivors were compared, survivors had higher APAP etiology rates (64.7% vs. 35.3%, *p* = 0.014), lower rates of fulfilling KCC (4.6% vs. 29.4%, *p* < 0.001), and had higher ALFSG-PI (68.1% vs. 31.3%, *p* < 0.001). Survivors required less life support in the form of mechanical ventilation (31.7% vs. 64.7%, *p* = 0.005), vasopressor use (11.3% vs. 31.6%, *p* < 0.001), and CRRT (9.0% vs. 23.5%, *p* = 0.047). Survivors developed fewer complications, including seizures (2.8% vs. 11.8%, *p* = 0.041), abnormal chest x-rays (44.5% vs. 100%, *p* = 0.004), and infections (9.5% vs. 29.4%, *p* = 0.009). Comparison between survivors and nonsurvivors of nonlisted (not sick enough) patients is demonstrated in Supplemental Table S4, http://links.lww.com/HC9/B72.

#### Comparison of nonlisted (too sick) survivors and nonsurvivors

Nonlisted patients with ALF deemed too sick had a 34.3% survival rate. When survivors and nonsurvivors were compared, survivors had lower rates of fulfilling KCC (15.3% vs. 39.9%, *p* < 0.001), lower MELD scores (36 vs. 41, *p* < 0.001), lower rates of high-grade HE (61.3% vs. 83.7%, *p* < 0.001), and higher ALFSG-PI (33.3% vs. 10.1%, *p* < 0.001). Survivors also required less life support in the form of mechanical ventilation (57.3% vs. 76.1%, *p* < 0.001) and vasopressor use (31.5% vs. 60.9%, *p* < 0.001). When looking at admission biochemistry, survivors had lower INR (2.3 vs. 3.2, *p* < 0.001), lower bilirubin (4.6 vs. 10.1, *p* < 0.001), lower ammonia (72 vs. 114, *p* < 0.001), and lower lactate (3.1 vs. 7.0, *p* < 0.001). Aside from having higher rates of infection, survivors and nonsurvivors had similar rates of all other complications. Comparison between survivors and nonsurvivors of nonlisted (too sick) patients is demonstrated in Supplemental Table S5, http://links.lww.com/HC9/B72.

## DISCUSSION

### Key results

In this study of 1672 patients with ALF not listed for LT, roughly a third fell into each of 3 categories: 33.3% of patients were deemed not sick enough, 28.4% of patients had psychosocial contraindications, and 29.8% of patients were deemed too sick to benefit from LT. Overall survival at 21 days was 64.3%. Overall 21-day survival was 95.8% for patients not listed due to being not sick enough and 34.3% for patients not listed due to being considered too sick to survive. After adjusting for covariates, factors associated with improved overall 21-day survival in nonlisted patients included being enrolled in the recent era, having APAP etiology for ALF, having a high ALFSG-PI, and using CRRT.

Nonlisted patients who were not sick enough were classified as such for a number of reasons. Close to 95% did not fulfill KCC, and over 60% had APAP as the etiology of ALF. A minority of these patients required life support measures. These patients had signs of ongoing clinical improvement early in their course. Nonlisted patients who were too sick were also classified as such for a number of reasons by the treating team. Having irreversible brain injury (1.6%) and sepsis (3%) were factors, along with the need for high amounts of life support (close to 70% required mechanical ventilation and over 50% required vasopressors), presence of MOF (cause of death in 30%), and having pre-existing conditions that would make surviving LT a challenge.

When comparing overall nonlisted patients to listed patients, nonlisted patients were more likely to have APAP etiology of ALF, less likely to require mechanical ventilation and RRT, less likely to meet KCC, have higher ALFSG-PI, more likely to develop bacteremia, and more likely to die. When compared to listed patients, nonlisted patients who were not sick enough were more likely to survive, while nonlisted patients who were too sick had lower rates of survival with higher rates of death by MOF. Overall, clinicians were more accurate in deeming patients not sick enough to need LT (>95% survival rate) as opposed to deeming patients too sick to survive (still >30% survival rate).

### Comparison with the literature

This study represents a large multicenter cohort of patients with ALF not listed for LT and provides a detailed assessment of outcomes and clinical and biochemical factors that affect these outcomes. Despite not being listed for LT, overall 21-day survival in this study was 64.3%, which is greater than previously reported TFS rates of 43%–56.2%.^15^,^21^ Over the past 20 years, outcomes have significantly improved for patients with ALF.^4^,^8^ In this study, there was an era effect seen with being enrolled between 2009 and 2018, which was independently associated with survival in nonlisted patients with ALF, suggesting improved outcomes over the years. This is similar to recent studies by Karvellas et al and MacDonald et al, showing improved survival over the years in patients with ALF listed for LT, as well as specifically patients with APAP-induced ALF.^4^,^8^ A major factor for improved survival is better prevention of cerebral edema through the early use of CRRT.^11^,^12^,^22^,^23^ The use of CRRT was also independently associated with survival in our study. The requirement of mechanical ventilation, which is most often for decreased mental status and airway protection, was independently associated with mortality in this study. However, 39% of patients with ALF require mechanical ventilation for significant lung injury, which negatively impacts outcomes.^24^

The etiology of ALF has been demonstrated to be an important factor in the prognosis of ALF. TFS was previously reported at 40% in APAP-related ALF compared with only 11% for non-APAP–related ALF.^25^ Similarly, having APAP as the etiology of ALF pertained to a better prognosis and was independently associated with survival in this study. In a study of patients with ALF listed for LT, patients with APAP etiology had spontaneous survival of 60% (more than twice that of non-APAP etiology) and were only transplanted 16% of the time.^16^

Ammonia levels are associated with complications and can affect outcomes in patients with ALF, even if severe hyperammonemia (>150 μmol/L) is not necessarily present. Previously, Bernal et al^26^ demonstrated that an ammonia level >100 μmol/L was associated with severe HE. Even with persistent ammonia levels >85 μmol/L, patients with ALF had an increased risk of complications and death.^27^ Our study found that nonlisted patients had a median ammonia level of 89 μmol/L, with those who died having a much higher median ammonia level of 112 μmol/L. The vast majority of nonlisted patients who died had severe HE (85.2%), which our study found was independently associated with death at 21 days.

Despite the majority of nonlisted patients with ALF surviving and with steady improvements in outcomes over the years, listing for LT is still often required for patients not responding to maximal medical therapies. This study demonstrated significantly higher 21-day overall survival rates in patients listed for LT (75.7% vs. 64.3%, *p* < 0.001) compared to nonlisted patients. This is most likely related to excellent survival after LT. Karvellas et al^4^ recently demonstrated one-year and 3-year post-LT survival rates of 91% and 90%, respectively. Previously, Reddy et al^16^ also showed excellent survival (92%) after LT in patients with ALF listed for LT. Also seen in this cohort, patients with ALF not ultimately transplanted had a spontaneous survival of 52%. LT, therefore, needs to be seriously considered in patients with ALF who show no signs of spontaneous recovery.

With improvements in outcomes, prognostication in ALF remains highly important. Prognostic tools such as KCC, MELD, and ALFSG-PI all independently affected overall 21-day mortality in this study, with meeting KCC and having high MELD associated with increased 21-day mortality while having high ALFSG-PI being associated with reduced 21-day mortality. Previous studies have shown KCC to have a sensitivity and specificity for predicting mortality of 58%–68% and 82%–95%, respectively, depending on whether ALF was APAP-induced or not.^28^,^29^ This suggests that KCC is better at predicting mortality than predicting survival. MELD was shown to have similar sensitivity and specificity for predicting mortality when compared with KCC.^30^ More recently, the ALFSG-PI was developed to help predict 21-day TFS as opposed to mortality and performed better than both KCC and MELD.^19^ However, the ALFSG-PI may overestimate the need for LT.^25^ Therefore, it appears prognostication still remains a challenge.

In this study, patients with ALF not listed for LT due to not being sick enough still had 4% mortality at 21 days, whereas patients deemed too sick to survive had 34.3% survival at 21 days. When looking at the entire group of nonlisted patients, fulfillment of KCC was lower, and ALFSG-PI was higher compared to listed patients. For the subcategory of nonlisted patients who were too well, fulfillment of KCC was also lower, and ALFSG-PI was also higher compared to listed patients. However, for the subcategory of nonlisted patients who were too sick, there was no difference in fulfillment of KCC and ALFSG-PI compared to listed patients. This suggests a better ability, although not perfect, to predict patients who are well and will spontaneously survive (>95% survival rate in nonlisted patients deemed not sick enough) as opposed to being able to predict patients who are too sick and will not survive (>30% survival in nonlisted patients deemed too sick). Further studies looking at prognostication in ALF are still needed.

### Strengths and limitations

This study should be interpreted in light of its strengths and limitations. The strengths include the recruitment of patients from multiple intensive care units across many geographic regions in North America. Patients in this study were mostly young, female, and demographically similar to patient populations reported in previous ALF studies from North America and Europe. Limitations include the retrospective nature of the analysis, resulting in only associations being drawn, and the inability to conclusively rule out sources of selection bias. Given that the ALFSG registry does not have complete clinical information before day 1 of study enrollment, we cannot exclude a possible referral bias of patients from referring hospitals to ALFSG enrolling sites. Data on patients enrolled in the ALFSG registry from the referring hospital (ie, pre-enrollment requirement for organ support) were unavailable. Development of the ALFSG-PI used patient data from the ALFSG registry, so many of the same patients were also included in this current study. Therefore, even with no collinearity seen in multivariate analysis, ALSFG-PI may not entirely be an independent variable, and its prognostic importance should be viewed within this limitation. Despite the limitations, this study is the most recent and largest cohort of consecutive patients with ALF not listed for transplant evaluating clinical outcomes across multiple tertiary care centers, allowing for broad generalizability of the results.

## CONCLUSIONS

Patients with ALF may not undergo listing for LT for a multitude of reasons. Despite this, the majority of these patients are still alive at 21 days, especially those with APAP as the etiology. When compared to patients listed for LT, nonlisted patients did have lower 21-day survival. Accurate prognostication is important in this patient population, and clinicians are highly accurate at predicting those who are not sick enough to need an LT (spontaneous survival rate of over 95%). Prognosticating patients deemed too sick to survive, however, still remains somewhat of a challenge (spontaneous survival rates of over 30%).

## Supplementary Material

SUPPLEMENTARY MATERIAL
