# Supplementary material for: Outcomes of patients with acute liver failure not listed for liver transplantation: A cohort analysis
Source: Hepatol Commun. 2024 Oct 30;8(11):e0575. doi: 10.1097/HC9.0000000000000575 (PMC11524736; doi:10.1097/HC9.0000000000000575)
Supplement: SUPPLEMENTARY MATERIAL [file hc9-8-e0575-s001.docx]

|  | **Not Listed** | | **Listed** | | **p-value** |
| --- | --- | --- | --- | --- | --- |
|  | N |  | N |  |  |
| **Age** | 1564 | 42 (30-54) | 934 | 38 (27-50) | <0.001 |
| **Year of enrollment** |  |  |  |  |  |
| 1998-2008 | 1564 | 760 (48.6%) | 934 | 570 (61.0%) | <0.001 |
| 2009-2018 | 1564 | 804 (51.4%) | 934 | 364 (39.0%) | <0.001 |
| **Sex (male)** | 1564 | 497 (31.8%) | 934 | 288 (30.8%) | 0.624 |
| **Etiology (we will get all the etiology information)** |  |  |  |  |  |
| **APAP** | 1564 | 843 (53.9%) | 934 | 272 (29.1%) | <0.001 |
| **DILI** | 1564 | 130 (8.3%) | 934 | 153 (16.4%) | <0.001 |
| **HBV** | 1564 | 80 (5.1%) | 934 | 105 (11.2%) | <0.001 |
| **AIH** | 1564 | 54 (3.5%) | 934 | 117 (12.5%) | <0.001 |
| **Indeterminate** | 1564 | 122 (7.8%) | 934 | 169 (18.1%) | <0.001 |
| **Other** | 1564 | 90 (5.8%) | 934 | 28 (3.0%) | 0.002 |
| **King’s College Criteria** | 1564 | 324 (20.7%) | 934 | 264 (28.3%) | <0.001 |
| **Highest MELD (during 7 days)** | 1550 | 36 (26-44) | 919 | 36 (30-42) | 0.290 |
| **Coma Grade 3/4 (highest during days 1-7)** | 1535 | 948 (61.8%) | 895 | 585 (65.4%) | 0.076 |
| **ALFSG prognostic index** | 1521 | 36.8% (12.4%-69.0%) | 883 | 15.0% (5.9%-31.7%) | <0.001 |
| **Organ Support (days 1-7)** |  |  |  |  |  |
| Mechanical Ventilation | 1564 | 905 (57.9%) | 934 | 600 (64.2%) | 0.002 |
| Vasopressors | 1564 | 540 (34.5%) | 934 | 308 (33.0%) | 0.429 |
| Renal Replacement Therapy | 1564 | 520 (33.3%) | 933 | 347 (37.2%) | 0.045 |
| Hemodialysis | 1564 | 334 (21.4%) | 934 | 252 (27.0%) | 0.001 |
| CVVH | 1564 | 244 (15.6%) | 933 | 126 (13.5%) | 0.154 |
| **Admission Biochemistry** |  |  |  |  |  |
| Hemoglobin (g/dL) | 1542 | 10.8 (9.4-12.3) | 921 | 11.2 (9.5-12.9) | <0.001 |
| White Blood Cells (10^9^/L) | 1541 | 9.8 (6.6-14.6) | 926 | 10.8 (7.3-15.4) | <0.001 |
| Platelets (10^9^/L) | 1534 | 119 (74-179) | 922 | 135 (90-200) | <0.001 |
| INR | 1526 | 2.6 (1.9-4.0) | 910 | 3 (2.3-4.5) | <0.001 |
| ALT (IU/L) | 1530 | 2344 (830-4620) | 923 | 1191 (384-3687) | <0.001 |
| Bilirubin (mg/dL) | 1532 | 5.3 (3.0-11.2) | 920 | 16.1 (6.2-26.0) | <0.001 |
| pH | 1152 | 7.41 (7.34-7.47) | 675 | 7.44 (7.38-7.49) | <0.001 |
| Ammonia (venous) (μmol/L) | 592 | 89 (58-140) | 301 | 107 (73-174) | <0.001 |
| Creatinine (mg/dL) | 1548 | 1.8 (0.9-3.2) | 928 | 1.30 (0.80-2.60) | <0.001 |
| Lactate (mmol/L) | 911 | 3.7 (2.2-7.2) | 442 | 4.9 (2.8-9.1) | <0.001 |
| Phosphate (mg/dL) | 1336 | 3 (2.0-4.4) | 778 | 3.2 (2.2-4.5) | 0.086 |
| **ICP therapies** |  |  |  |  |  |
| ICP monitor | 1474 | 79 (5.4%) | 813 | 208 (25.6%) | <0.001 |
| Mannitol | 1564 | 197 (12.6%) | 934 | 234 (25.1%) | <0.001 |
| Barbiturate | 1564 | 76 (4.9%) | 934 | 83 (8.9%) | <0.001 |
| Hypothermia | 1564 | 71 (4.5%) | 933 | 57 (6.1%) | 0.085 |
| Sedatives | 1564 | 902 (57.7%) | 934 | 549 (58.8%) | 0.588 |
| **Blood Products (days 1-7)** |  |  |  |  |  |
| Red Blood Cells | 1564 | 433 (27.7%) | 934 | 390 (41.8%) | <0.001 |
| Fresh Frozen Plasma | 1564 | 640 (40.9%) | 934 | 637 (68.2%) | <0.001 |
| Platelets | 1564 | 272 (17.4%) | 934 | 254 (27.2%) | <0.001 |
| **ICU Complications (days 1-7)** |  |  |  |  |  |
| Seizures | 1564 | 86 (5.5%) | 934 | 51 (5.5%) | 0.968 |
| Arrhythmia | 1564 | 340 (21.7%) | 934 | 188 (20.1%) | 0.340 |
| Gastrointestinal Bleeding | 1564 | 144 (9.2%) | 934 | 67 (7.2%) | 0.077 |
| Abnormal Chest X-Ray | 844 | 556 (65.9%) | 847 | 536 (63.3%) | 0.265 |
| Bacteremia/Blood Stream Infection | 1564 | 227 (14.5%) | 933 | 53 (5.7%) | <0.001 |
| **Intra-Study NAC** |  |  |  |  |  |
| IV | 1564 | 1029 (65.8%) | 934 | 415 (44.4%) | <0.001 |
| Oral | 1564 | 658 (42.1%) | 934 | 313 (33.5%) | <0.001 |
| **Psychiatric Comorbidities** | 1564 | 645 (41.2%) | 934 | 202 (21.63%) | <0.001 |
| **Overdose Intent (IF APAP)** |  |  |  |  |  |
| Suicide Attempt | 897 | 327 (36.5%) | 315 | 97 (30.8%) | 0.070 |
| Unintentional | 897 | 454 (50.6%) | 315 | 164 (52.1%) | 0.658 |
| Unknown | 897 | 116 (12.9%) | 315 | 54 (17.1%) | 0.064 |
| **Alcohol Use** |  |  |  |  |  |
| <7 drinks/week | 638 | 463 (72.6%) | 278 | 235 (84.5%) | <0.001 |
| >=7 drinks/week | 638 | 175 (27.4%) | 278 | 43 (15.5%) | <0.001 |
| **Intravenous Drug Use** | 1549 | 119 (7.7%) | 923 | 21 (2.3%) | <0.001 |
| **Death (days 1-21)** | 1564 | 558 (35.7%) | 845 | 205 (24.3%) | <0.001 |
| **Cause of Death** |  |  |  |  |  |
| Multi-Organ Failure | 1297 | 235 (18.1%) | 635 | 68 (10.7%) | <0.001 |
| Cerebral Edema | 1297 | 80 (6.2%) | 635 | 66 (10.4%) | <0.001 |
| Unknown | 1297 | 84 (6.5%) | 635 | 20 (3.2%) | 0.002 |
| **Outcome (day 21)** |  |  |  |  |  |
| Survival | 1,564 | 1006 (64.3%) | 845 | 640 (75.7%) | <0.001 |
| Death | 1,564 | 558 (35.7%) | 845 | 205 (24.3%) | <0.001 |

**Supplementary Table 1. Demographics and outcomes of 2498 ALF patients stratified by listing or non-listing for LT**Abbreviations: ALF, acute liver failure; LT, liver transplantation; APAP, acetaminophen; DILI, drug-induced liver injury; HBV, hepatitis B virus; AIH, autoimmune hepatitis; MELD, model for end-stage liver disease; ALFSG, Acute Liver Failure Study Group; CVVH, continuous veno-venous hemofiltration; INR, international normalized ratio; ALT, alanine transaminase; ICP, intracranial pressure; IV, intravenous

|  | **Not Listed – Too Well** | | **Listed** | | **p-value** |
| --- | --- | --- | --- | --- | --- |
|  | N |  | N |  |  |
| **Age** | 443 | 40 (29-52) | 934 | 38 (27-50) | 0.076 |
| **Year of enrollment** |  |  |  |  |  |
| 1998-2008 | 443 | 172 (38.8%) | 934 | 570 (61.0%) | <0.001 |
| 2009-2018 | 443 | 271 (61.2%) | 934 | 364 (39.0%) | <0.001 |
| **Sex (male)** | 443 | 108 (24.4%) | 934 | 288 (30.8%) | 0.013 |
| **Etiology (we will get all the etiology information)** |  |  |  |  |  |
| **APAP** | 443 | 282 (63.7%) | 934 | 272 (29.1%) | <0.001 |
| **DILI** | 443 | 31 (7.0%) | 934 | 153 (16.4%) | <0.001 |
| **HBV** | 443 | 18 (4.1%) | 934 | 105 (11.2%) | <0.001 |
| **AIH** | 443 | 8 (1.8%) | 934 | 117 (12.5%) | <0.001 |
| **Indeterminate** | 443 | 27 (6.1%) | 934 | 169 (18.1%) | <0.001 |
| **Other** | 443 | 9 (2.0%) | 934 | 28 (3.0%) | 0.300 |
| **King’s College Criteria** | 443 | 24 (5.4%) | 934 | 264 (28.3%) | <0.001 |
| **Highest MELD (during 7 days)** | 443 | 29 (20-37) | 919 | 36 (30-42) | <0.001 |
| **Coma Grade 3/4 (highest during days 1-7)** | 433 | 148 (34.2%) | 895 | 585 (65.4%) | <0.001 |
| **ALFSG prognostic index** | 433 | 67.6% (39.6%-86.3%) | 883 | 15.0% (5.9%-31.7%) | <0.001 |
| **Organ Support (days 1-7)** |  |  |  |  |  |
| Mechanical Ventilation | 443 | 154 (34.8%) | 934 | 600 (64.2%) | <0.001 |
| Vasopressors | 443 | 64 (14.4%) | 934 | 308 (33.0%) | <0.001 |
| Renal Replacement Therapy | 443 | 91 (20.5%) | 933 | 347 (37.2%) | <0.001 |
| Hemodialysis | 443 | 62 (14.0%) | 934 | 252 (27.0%) | <0.001 |
| CVVH | 443 | 39 (8.8%) | 933 | 126 (13.5%) | 0.012 |
| **Admission Biochemistry** |  |  |  |  |  |
| Hemoglobin (g/dL) | 438 | 11.1 (9.7-12.5) | 921 | 11.2 (9.5-12.9) | 0.327 |
| White Blood Cells (10^9^/L) | 437 | 9.2 (6.4-12.8) | 926 | 10.8 (7.3-15.4) | <0.001 |
| Platelets (10^9^/L) | 435 | 137 (93-192) | 922 | 135 (90-200) | 0.985 |
| INR | 434 | 2.2 (1.8-3.1) | 910 | 3 (2.3-4.5) | <0.001 |
| ALT (IU/L) | 438 | 2702 (1304-4807) | 923 | 1191 (384-3687) | <0.001 |
| Bilirubin (mg/dL) | 435 | 4 (2.1-6.8) | 920 | 16.1 (6.2-26.0) | <0.001 |
| pH | 295 | 7.42 (7.37-7.48) | 675 | 7.44 (7.38-7.49) | 0.002 |
| Ammonia (venous) (μmol/L) | 206 | 76 (55-113) | 301 | 107 (73-174) | <0.001 |
| Creatinine (mg/dL) | 440 | 1.30 (0.71-2.85) | 928 | 1.30 (0.80-2.60) | 0.307 |
| Lactate (mmol/L) | 257 | 2.4 (1.5-3.9) | 442 | 4.9 (2.8-9.1) | <0.001 |
| Phosphate (mg/dL) | 380 | 2.5 (1.8-3.5) | 778 | 3.2 (2.2-4.5) | <0.001 |
| **ICP therapies** |  |  |  |  |  |
| ICP monitor | 434 | 10 (2.3%) | 813 | 208 (25.6%) | <0.001 |
| Mannitol | 443 | 26 (5.9%) | 934 | 234 (25.1%) | <0.001 |
| Barbiturate | 443 | 14 (3.2%) | 934 | 83 (8.9%) | <0.001 |
| Hypothermia | 443 | 10 (2.3%) | 933 | 57 (6.1%) | 0.002 |
| Sedatives | 443 | 178 (40.2%) | 934 | 549 (58.8%) | <0.001 |
| **Blood Products (days 1-7)** |  |  |  |  |  |
| Red Blood Cells | 443 | 76 (17.2%) | 934 | 390 (41.8%) | <0.001 |
| Fresh Frozen Plasma | 443 | 111 (25.1%) | 934 | 637 (68.2%) | <0.001 |
| Platelets | 443 | 32 (7.2%) | 934 | 254 (27.2%) | <0.001 |
| **ICU Complications (days 1-7)** |  |  |  |  |  |
| Seizures | 443 | 14 (3.2%) | 934 | 51 (5.5%) | 0.060 |
| Arrhythmia | 443 | 50 (11.3%) | 934 | 188 (20.1%) | <0.001 |
| Gastrointestinal Bleeding | 443 | 22 (5.0%) | 934 | 67 (7.2%) | 0.120 |
| Abnormal Chest X-Ray | 163 | 81 (49.7%) | 847 | 536 (63.3%) | 0.001 |
| Bacteremia/Blood Stream Infection | 443 | 42 (9.5%) | 933 | 53 (5.7%) | 0.009 |
| **Intra-Study NAC** |  |  |  |  |  |
| IV | 443 | 309 (69.8%) | 934 | 415 (44.4%) | <0.001 |
| Oral | 443 | 219 (49.4%) | 934 | 313 (33.5%) | <0.001 |
| **Psychiatric Comorbidities** | 443 | 212 (47.9%) | 934 | 202 (21.63%) | <0.001 |
| **Overdose Intent (IF APAP)** |  |  |  |  |  |
| Suicide Attempt | 284 | 83 (29.2%) | 315 | 97 (30.8%) | 0.676 |
| Unintentional | 284 | 173 (60.9%) | 315 | 164 (52.1%) | 0.029 |
| Unknown | 284 | 28 (9.9%) | 315 | 54 (17.1%) | 0.010 |
| **Alcohol Use** |  |  |  |  |  |
| <7 drinks/week | 202 | 148 (73.3%) | 278 | 235 (84.5%) | 0.002 |
| >=7 drinks/week | 202 | 54 (26.7%) | 278 | 43 (15.5%) | 0.002 |
| **Intravenous Drug Use** | 440 | 31 (7.0%) | 923 | 21 (2.3%) | <0.001 |
| **Death (days 1-21)** | 405 | 17 (4.2%) | 845 | 205 (24.3%) | <0.001 |
| **Cause of Death** |  |  |  |  |  |
| Multi-Organ Failure | 325 | 6 (1.8%) | 635 | 68 (10.7%) | <0.001 |
| Cerebral Edema | 325 | 0 (0.0%) | 635 | 66 (10.4%) | <0.001 |
| Unknown | 325 | 14 (4.3%) | 635 | 20 (3.2%) | 0.358 |
| **Outcome (day 21)** |  |  |  |  |  |
| Survival | 405 | 388 (95.8%) | 845 | 640 (75.7%) | <0.001 |
| Death | 405 | 17 (4.2%) | 845 | 205 (24.3%) | <0.001 |

**Supplementary Table 2. Demographics and outcomes of 1377 ALF patients stratified by LT listing or non-listing due to being too well**
Abbreviations: ALF, acute liver failure; LT, liver transplantation; APAP, acetaminophen; DILI, drug-induced liver injury; HBV, hepatitis B virus; AIH, autoimmune hepatitis; MELD, model for end-stage liver disease; ALFSG, Acute Liver Failure Study Group; CVVH, continuous veno-venous hemofiltration; INR, international normalized ratio; ALT, alanine transaminase; ICP, intracranial pressure; IV, intravenous

|  | **Not Listed – Too Sick** | | **Listed** | | **p-value** |
| --- | --- | --- | --- | --- | --- |
|  | N |  | N |  |  |
| **Age** | 371 | 51 (38-63) | 934 | 38 (27-50) | <0.001 |
| **Year of enrollment** |  |  |  |  |  |
| 1998-2008 | 371 | 110 (29.6%) | 934 | 570 (61.0%) | <0.001 |
| 2009-2018 | 371 | 261 (70.4%) | 934 | 364 (39.0%) | <0.001 |
| **Sex (male)** | 371 | 147 (39.6%) | 934 | 288 (30.8%) | 0.002 |
| **Etiology (we will get all the etiology information)** |  |  |  |  |  |
| **APAP** | 371 | 91 (24.5%) | 934 | 272 (29.1%) | 0.095 |
| **DILI** | 371 | 45 (12.1%) | 934 | 153 (16.4%) | 0.054 |
| **HBV** | 371 | 27 (7.3%) | 934 | 105 (11.2%) | 0.032 |
| **AIH** | 371 | 18 (4.9%) | 934 | 117 (12.5%) | <0.001 |
| **Indeterminate** | 371 | 36 (9.7%) | 934 | 169 (18.1%) | <0.001 |
| **Other** | 371 | 51 (13.7%) | 934 | 28 (3.0%) | <0.001 |
| **King’s College Criteria** | 371 | 114 (30.7%) | 934 | 264 (28.3%) | 0.376 |
| **Highest MELD (during 7 days)** | 365 | 40 (33-47) | 919 | 36 (30-42) | <0.001 |
| **Coma Grade 3/4 (highest during days 1-7)** | 364 | 274 (75.3%) | 895 | 585 (65.4%) | <0.001 |
| **ALFSG prognostic index** | 358 | 15.4% (6.1%-37.0%) | 883 | 15.0% (5.9%-31.7%) | 0.609 |
| **Organ Support (days 1-7)** |  |  |  |  |  |
| Mechanical Ventilation | 371 | 258 (69.5%) | 934 | 600 (64.2%) | 0.069 |
| Vasopressors | 371 | 189 (50.9%) | 934 | 308 (33.0%) | <0.001 |
| Renal Replacement Therapy | 371 | 158 (42.6%) | 933 | 347 (37.2%) | 0.071 |
| Hemodialysis | 371 | 96 (25.9%) | 934 | 252 (27.0%) | 0.684 |
| CVVH | 371 | 72 (19.4%) | 933 | 126 (13.5%) | 0.007 |
| **Admission Biochemistry** |  |  |  |  |  |
| Hemoglobin (g/dL) | 368 | 10.1 (8.8-11.6) | 921 | 11.2 (9.5-12.9) | <0.001 |
| White Blood Cells (10^9^/L) | 368 | 11.4 (7.5-16.5) | 926 | 10.8 (7.3-15.4) | 0.426 |
| Platelets (10^9^/L) | 365 | 100 (58-151) | 922 | 135 (90-200) | <0.001 |
| INR | 360 | 2.8 (2.1-4.3) | 910 | 3 (2.3-4.5) | 0.017 |
| ALT (IU/L) | 362 | 1406 (497-3430) | 923 | 1191 (384-3687) | 0.570 |
| Bilirubin (mg/dL) | 363 | 7.2 (3.3-18.1) | 920 | 16.1 (6.2-26.0) | <0.001 |
| pH | 273 | 7.39 (7.30-7.45) | 675 | 7.44 (7.38-7.49) | <0.001 |
| Ammonia (venous) (μmol/L) | 155 | 90 (60-156) | 301 | 107 (73-174) | 0.016 |
| Creatinine (mg/dL) | 367 | 2.2 (1.3-3.6) | 928 | 1.30 (0.80-2.60) | <0.001 |
| Lactate (mmol/L) | 221 | 4.9 (2.8-9.8) | 442 | 4.9 (2.8-9.1) | 0.663 |
| Phosphate (mg/dL) | 316 | 3.9 (2.8-5.5) | 778 | 3.2 (2.2-4.5) | <0.001 |
| **ICP therapies** |  |  |  |  |  |
| ICP monitor | 369 | 18 (4.9%) | 813 | 208 (25.6%) | <0.001 |
| Mannitol | 371 | 45 (12.1%) | 934 | 234 (25.1%) | <0.001 |
| Barbiturate | 371 | 15 (4.0%) | 934 | 83 (8.9%) | 0.003 |
| Hypothermia | 371 | 26 (7.0%) | 933 | 57 (6.1%) | 0.549 |
| Sedatives | 371 | 227 (61.2%) | 934 | 549 (58.8%) | 0.424 |
| **Blood Products (days 1-7)** |  |  |  |  |  |
| Red Blood Cells | 371 | 120 (32.3%) | 934 | 390 (41.8%) | 0.002 |
| Fresh Frozen Plasma | 371 | 167 (45.0%) | 934 | 637 (68.2%) | <0.001 |
| Platelets | 371 | 91 (24.5%) | 934 | 254 (27.2%) | 0.325 |
| **ICU Complications (days 1-7)** |  |  |  |  |  |
| Seizures | 371 | 20 (5.4%) | 934 | 51 (5.5%) | 0.960 |
| Arrhythmia | 371 | 100 (27.0%) | 934 | 188 (20.1%) | 0.007 |
| Gastrointestinal Bleeding | 371 | 35 (9.4%) | 934 | 67 (7.2%) | 0.170 |
| Abnormal Chest X-Ray | 176 | 135 (76.7%) | 847 | 536 (63.3%) | <0.001 |
| Bacteremia/Blood Stream Infection | 371 | 94 (25.3%) | 933 | 53 (5.7%) | <0.001 |
| **Intra-Study NAC** |  |  |  |  |  |
| IV | 371 | 222 (59.8%) | 934 | 415 (44.4%) | <0.001 |
| Oral | 371 | 94 (25.3%) | 934 | 313 (33.5%) | 0.004 |
| **Psychological Comorbidities** | 371 | 103 (27.8%) | 934 | 202 (21.63%) | 0.018 |
| **Overdose Intent (IF APAP)** |  |  |  |  |  |
| Suicide Attempt | 105 | 28 (26.7%) | 315 | 97 (30.8%) | 0.423 |
| Unintentional | 105 | 58 (55.2%) | 315 | 164 (52.1%) | 0.573 |
| Unknown | 105 | 19 (18.1%) | 315 | 54 (17.1%) | 0.824 |
| **Alcohol Use** |  |  |  |  |  |
| <7 drinks/week | 208 | 176 (84.6%) | 278 | 235 (84.5%) | 0.980 |
| >=7 drinks/week | 208 | 32 (15.4%) | 278 | 43 (15.5%) | 0.980 |
| **Intravenous Drug Use** | 367 | 16 (4.4%) | 923 | 21 (2.3%) | 0.043 |
| **Death (days 1-21)** | 362 | 238 (65.7%) | 845 | 205 (24.3%) | <0.001 |
| **Cause of Death** |  |  |  |  |  |
| Multi-Organ Failure | 354 | 107 (30.2%) | 635 | 68 (10.7%) | <0.001 |
| Cerebral Edema | 354 | 26 (7.3%) | 635 | 66 (10.4%) | 0.114 |
| Unknown | 354 | 23 (6.5%) | 635 | 20 (3.2%) | 0.013 |
| **Outcome (day 21)** |  |  |  |  |  |
| Survival | 362 | 124 (34.3%) | 845 | 640 (75.7%) | <0.001 |
| Death | 362 | 238 (65.7%) | 845 | 205 (24.3%) | <0.001 |

**Supplementary Table 3. Demographics and outcomes of 1305 ALF patients stratified by LT listing or non-listing due to being too sick**
Abbreviations: ALF, acute liver failure; LT, liver transplantation; APAP, acetaminophen; DILI, drug-induced liver injury; HBV, hepatitis B virus; AIH, autoimmune hepatitis; MELD, model for end-stage liver disease; ALFSG, Acute Liver Failure Study Group; CVVH, continuous veno-venous hemofiltration; INR, international normalized ratio; ALT, alanine transaminase; ICP, intracranial pressure; IV, intravenous

| **Not Listed – Too Well** | **Alive at day 21 (N = 388)** | | **Deceased at day 21 (N=17)** | | **p-value** |
| --- | --- | --- | --- | --- | --- |
|  | N |  | N |  |  |
| **Age** | 388 | 40 (29-52) | 17 | 45 (37-61) | 0.075 |
| **Year of enrollment** | 388 |  | 17 |  |  |
| 1998 to 2008 | 388 | 148 (38.1%) | 17 | 5 (29.4%) | 0.467 |
| 2009 to 2018 | 388 | 240 (61.9%) | 17 | 12 (70.6%) | 0.467 |
| **Sex (male)** | 388 | 98 (25.3%) | 17 | 3 (17.7%) | 0.478 |
| **Etiology (we will get all the etiology information)** |  |  |  |  |  |
| **APAP** | 388 | 251 (64.7%) | 17 | 6 (35.3%) | 0.014 |
| **DILI** | 388 | 29 (7.5%) | 17 | 1 (5.9%) | 0.806 |
| **HBV** | 388 | 15 (3.9%) | 17 | 2 (11.8%) | 0.112 |
| **AIH** | 388 | 8 (2.1%) | 17 | 0 (0.0%) | 0.550 |
| **Indeterminate** | 388 | 21 (5.4%) | 17 | 2 (11.8%) | 0.268 |
| **Other** | 388 | 4 (1.0%) | 17 | 2 (11.8%) | <0.001 |
| **King’s College Criteria** | 388 | 18 (4.6%) | 17 | 5 (29.4%) | <0.001 |
| **Highest MELD (during 7 days)** | 388 | 29 (20-37) | 17 | 34 (25-38) | 0.171 |
| **Coma Grade 3/4 (highest during days 1-7)** | 388 | 124 (32.8%) | 17 | 8 (47.1%) | 0.223 |
| **ALFSG Prognostic Index (highest days 1-7)** | 388 | 68.1% (41.0%-86.3%) | 17 | 31.3% (13.7%-49.9%) | <0.001 |
| **Organ Support (days 1-7)** |  |  |  |  |  |
| Mechanical Ventilation | 388 | 123 (31.7%) | 17 | 11 (64.7%) | 0.005 |
| Vasopressors | 388 | 44 (11.3%) | 17 | 8 (31.6%) | <0.001 |
| Renal Replacement Therapy | 388 | 77 (19.9%) | 17 | 5 (29.4%) | 0.337 |
| Hemodialysis | 388 | 51 (13.1%) | 17 | 2 (11.8%) | 0.869 |
| CVVH | 388 | 35 (9.0%) | 17 | 4 (23.5%) | 0.047 |
| **Admission Biochemistry** |  |  |  |  |  |
| Hemoglobin (g/dL) | 383 | 11.1 (9.7-12.6) | 17 | 10.0 (8.3-11.9) | 0.132 |
| White Blood Cells (10^9^/L) | 382 | 9.2 (6.4-12.6) | 17 | 14.1 (9.0-19.1) | 0.014 |
| Platelets (10^9^/L) | 380 | 141 (101-194) | 17 | 93 (57-192) | 0.091 |
| INR | 381 | 2.3 (1.8-3.2) | 16 | 2.6 (1.9-3.4) | 0.689 |
| ALT (IU/L) | 383 | 2767 (1414-4867) | 17 | 661 (328-1288) | <0.001 |
| Bilirubin (mg/dL) | 380 | 4.0 (2.1-6.8) | 17 | 4.7 (3.0-10.3) | 0.410 |
| pH | 258 | 7.42 (7.37-7.48) | 8 | 7.44 (7.35-7.49) | 0.779 |
| Ammonia (venous) (μmol/L) | 184 | 78 (53-115) | 5 | 62 (59-74) | 0.342 |
| Creatinine (mg/dL) | 385 | 1.3 (0.7-2.8) | 17 | 1.6 (1.0-2.4) | 0.659 |
| Lactate (mmol/L) | 227 | 2.3 (1.5-3.7) | 9 | 5.6 (2.5-15.0) | 0.005 |
| Phosphate (mg/dL) | 332 | 2.5 (1.7-3.5) | 14 | 3.0 (2.0-3.7) | 0.248 |
| **ICP therapies (days 1-7)** |  |  |  |  |  |
| ICP monitor | 379 | 9 (2.4%) | 17 | 0 (0.0%) | 0.520 |
| Mannitol | 388 | 19 (4.9%) | 17 | 2 (11.8%) | 0.211 |
| Barbiturate | 388 | 10 (2.6%) | 17 | 1 (5.9%) | 0.412 |
| Hypothermia | 388 | 8 (2.1%) | 17 | 0 (0.0%) | 0.550 |
| Sedatives | 388 | 150 (38.7%) | 17 | 8 (47.1%) | 0.487 |
| **Blood Products (days 1-7)** |  |  |  |  |  |
| Red Blood Cells | 388 | 62 (16.0%) | 17 | 7 (41.2%) | 0.007 |
| Fresh Frozen Plasma | 388 | 93 (24.0%) | 17 | 7 (41.2%) | 0.107 |
| Platelets | 388 | 28 (7.2%) | 17 | 2 (11.8%) | 0.483 |
| **ICU Complications (days 1-7)** |  |  |  |  |  |
| Seizures | 388 | 11 (2.8%) | 17 | 2 (11.8%) | 0.041 |
| Arrhythmia | 388 | 46 (11.7%) | 17 | 3 (17.7%) | 0.474 |
| Gastrointestinal Bleeding | 388 | 17 (4.4%) | 17 | 2 (11.8%) | 0.159 |
| Abnormal Chest X-Ray | 137 | 61 (44.5%) | 7 | 7 (100%) | 0.004 |
| Bacteremia/Blood Stream Infection | 388 | 37 (9.5%) | 17 | 5 (29.4%) | 0.009 |
| **Intra-Study NAC** |  |  |  |  |  |
| IV | 388 | 268 (69.1%) | 17 | 12 (70.6%) | 0.895 |
| Oral | 388 | 184 (47.4%) | 17 | 7 (41.2%) | 0.614 |
| **Psychiatric Comorbidities** | 388 | 182 (46.9%) | 17 | 12 (70.6%) | 0.056 |
| **Overdose Intent (IF APAP)** |  |  |  |  |  |
| Suicide Attempt | 252 | 274 (29.4%) | 7 | 1 (14.3%) | 0.386 |
| Unintentional | 252 | 156 (61.9%) | 7 | 4 (57.1% | 0.798 |
| Unknown | 252 | 22 (8.7%) | 7 | 2 (28.6%) | 0.074 |
| **Alcohol Use** |  |  |  |  |  |
| <7 drinks/week | 190 | 141 (74.2%) | 11 | 7 (63.6%) | 0.439 |
| >=7 drinks/week | 190 | 49 (25.8%) | 11 | 4 (36.3%) | 0.439 |
| **Intravenous Drug Use** | 387 | 29 (7.5%) | 17 | 1 (5.9%) | 0.804 |

**Supplementary Table 4. Demographics of 405 ALF patients not listed for LT due to being too well stratified based on overall 21 day survival**Abbreviations: ALF, acute liver failure; LT, liver transplantation; APAP, acetaminophen; DILI, drug-induced liver injury; HBV, hepatitis B virus; AIH, autoimmune hepatitis; MELD, model for end-stage liver disease; ALFSG, Acute Liver Failure Study Group; CVVH, continuous veno-venous hemofiltration; INR, international normalized ratio; ALT, alanine transaminase; ICP, intracranial pressure; IV, intravenous

| **Not Listed – Too Sick** | **Alive at day 21 (N = 124)** | | **Deceased at day 21 (N=238)** | | **p-value** |
| --- | --- | --- | --- | --- | --- |
|  | N |  | N |  |  |
| **Age** | 124 | 50 (36-63) | 238 | 52 (39-63) | 0.624 |
| **Year of enrollment** | 124 |  | 238 |  |  |
| 1998 to 2008 | 124 | 26 (30.0%) | 238 | 77 (32.4%) | 0.023 |
| 2009 to 2018 | 124 | 98 (79.0%) | 238 | 161 (67.7%) | 0.023 |
| **Sex (male)** | 124 | 42 (33.9%) | 238 | 101 (42.4%) | 0.114 |
| **Etiology (we will get all the etiology information)** |  |  |  |  |  |
| **APAP** | 124 | 31 (25.0%) | 238 | 58 (24.4%) | 0.895 |
| **DILI** | 124 | 17 (13.7%) | 238 | 27 (11.3%) | 0.513 |
| **HBV** | 124 | 2 (1.6%) | 238 | 25 (10.5%) | 0.002 |
| **AIH** | 124 | 2 (1.6%) | 238 | 15 (6.3%) | 0.045 |
| **Indeterminate** | 124 | 10 (8.1%) | 238 | 26 (10.9%) | 0.388 |
| **Other** | 124 | 9 (7.3%) | 238 | 42 (17.6%) | 0.007 |
| **King’s College Criteria** | 124 | 19 (15.3%) | 238 | 95 (39.9%) | <0.001 |
| **Highest MELD (during 7 days)** | 124 | 36 (28-42) | 232 | 41 (34-48) | <0.001 |
| **Coma Grade 3/4 (highest during days 1-7)** | 124 | 76 (61.3%) | 233 | 195 (83.7%) | <0.001 |
| **ALFSG Prognostic Index (highest days 1-7)** | 124 | 33.3% (15.9%-54.4%) | 227 | 10.1% (3.7%-23.1%) | <0.001 |
| **Organ Support (days 1-7)** |  |  |  |  |  |
| Mechanical Ventilation | 124 | 71 (57.3%) | 238 | 181 (76.1%) | <0.001 |
| Vasopressors | 124 | 39 (31.5%) | 238 | 145 (60.9%) | <0.001 |
| Renal Replacement Therapy | 124 | 51 (41.1%) | 238 | 105 (44.1%) | 0.586 |
| Hemodialysis | 124 | 36 (29.0%) | 238 | 58 (24.4%) | 0.337 |
| CVVH | 124 | 21 (16.9%) | 238 | 51 (21.4%) | 0.310 |
| **Admission Biochemistry** |  |  |  |  |  |
| Hemoglobin (g/dL) | 122 | 10.0 (8.9-11.6) | 237 | 10.2 (8.7-11.6) | 0.620 |
| White Blood Cells (10^9^/L) | 123 | 12.4 (7.6-16.7) | 236 | 10.7 (7.2-16.4) | 0.375 |
| Platelets (10^9^/L) | 121 | 101 (53-152) | 235 | 97 (61-151) | 0.972 |
| INR | 121 | 2.3 (1.9-3.5) | 230 | 3.2 (2.3-4.6) | <0.001 |
| ALT (IU/L) | 122 | 2100 (783-3991) | 231 | 1165 (420-2936) | 0.002 |
| Bilirubin (mg/dL) | 122 | 4.6 (2.1-10.2) | 232 | 10.1 (4.3-21.5) | <0.001 |
| pH | 90 | 7.40 (7.34-7.44) | 175 | 7.38 (7.27-7.46) | 0.271 |
| Ammonia (venous) (μmol/L) | 53 | 72 (47-104) | 101 | 114 (71-171) | <0.001 |
| Creatinine (mg/dL) | 123 | 2.3 (1.2-3.6) | 235 | 2.2 (1.4-3.6) | 0.751 |
| Lactate (mmol/L) | 70 | 3.1 (2.2-5.1) | 147 | 7.0 (3.8-11.4) | <0.001 |
| Phosphate (mg/dL) | 104 | 3.3 (2.6-4.5) | 205 | 4.4 (3.0-5.9) | <0.001 |
| **ICP therapies (days 1-7)** |  |  |  |  |  |
| ICP monitor | 124 | 5 (4.0%) | 236 | 13 (5.5%) | 0.541 |
| Mannitol | 124 | 6 (4.8%) | 238 | 39 (16.4%) | 0.002 |
| Barbiturate | 124 | 1 (0.8%) | 238 | 14 (5.9%) | 0.022 |
| Hypothermia | 124 | 6 (4.8%) | 238 | 20 (8.4%) | 0.213 |
| Sedatives | 124 | 69 (55.7%) | 238 | 154 (64.7%) | 0.093 |
| **Blood Products (days 1-7)** |  |  |  |  |  |
| Red Blood Cells | 124 | 38 (30.7%) | 238 | 80 (33.6%) | 0.568 |
| Fresh Frozen Plasma | 124 | 39 (31.5%) | 238 | 125 (52.5%) | <0.001 |
| Platelets | 124 | 26 (21.0%) | 238 | 64 (26.9%) | 0.216 |
| **ICU Complications (days 1-7)** |  |  |  |  |  |
| Seizures | 124 | 4 (3.2%) | 238 | 16 (6.7%) | 0.167 |
| Arrhythmia | 124 | 34 (27.4%) | 238 | 63 (26.5%) | 0.847 |
| Gastrointestinal Bleeding | 124 | 9 (7.3%) | 238 | 26 (10.9%) | 0.263 |
| Abnormal Chest X-Ray | 38 | 29 (76.3%) | 134 | 102 (76.1%) | 0.980 |
| Bacteremia/Blood Stream Infection | 124 | 44 (35.5%) | 238 | 50 (21.0%) | 0.003 |
| **Intra-Study NAC** |  |  |  |  |  |
| IV | 124 | 78 (62.9%) | 238 | 140 (58.8%) | 0.576 |
| Oral | 124 | 27 (21.8%) | 238 | 63 (26.5%) | 0.327 |
| **Psychiatric Comorbidities** | 124 | 39 (31.5%) | 238 | 58 (24.4%) | 0.149 |
| **Overdose Intent (IF APAP)** |  |  |  |  |  |
| Suicide Attempt | 36 | 8 (22.2%) | 66 | 18 (27.3%) | 0.386 |
| Unintentional | 36 | 23 (63.9%) | 66 | 34 (51.5% | 0.229 |
| Unknown | 36 | 5 (13.9%) | 66 | 14 (21.2%) | 0.364 |
| **Alcohol Use** |  |  |  |  |  |
| <7 drinks/week | 82 | 70 (85.4%) | 126 | 106 (84.1%) | 0.809 |
| >=7 drinks/week | 82 | 12 (14.6%) | 126 | 20 (15.9%) | 0.809 |
| **Intravenous Drug Use** | 124 | 8 (6.5%) | 234 | 7 (3.0%) | 0.120 |

**Supplementary Table 5. Demographics of 362 ALF patients not listed for LT due to being too sick stratified based on overall 21 day survival**Abbreviations: ALF, acute liver failure; LT, liver transplantation; APAP, acetaminophen; DILI, drug-induced liver injury; HBV, hepatitis B virus; AIH, autoimmune hepatitis; MELD, model for end-stage liver disease; ALFSG, Acute Liver Failure Study Group; CVVH, continuous veno-venous hemofiltration; INR, international normalized ratio; ALT, alanine transaminase; ICP, intracranial pressure; IV, intravenous
